# Pictograms to Provide a Better Understanding of Gastroesophageal Reflux Symptoms in Chinese Subjects

**DOI:** 10.1155/2017/1214584

**Published:** 2017-06-01

**Authors:** Wei Zhao, Hong Jin, Lili Zhang, Bin Wang, Fangyuan Sun, Huanli Jiao, Chao Sun, Bangmao Wang

**Affiliations:** ^1^Department of GI, Tianjin Medical University General Hospital, Tianjin 300054, China; ^2^Physical Examination Center, Tianjin Medical University General Hospital, Tianjin 300054, China

## Abstract

**Objective:**

To explore whether pictograms could help people understand reflux symptoms.

**Methods:**

Gastroenterologists (*n* = 28), non-GI physicians (*n* = 30), healthy people without medical education (*n* = 34), patients with gastrointestinal reflux disease (GERD) (*n* = 45), and general people (*n* = 100) were included. Pictograms denoting classic reflux symptoms (sour regurgitation, heartburn, retrosternal pain, and regurgitation) were created by the joint efforts of an artist and a gastroenterologist. The subjects were asked to tell the meaning of each card within 30 s.

**Results:**

Compared with the physicians, healthy people without medical education tended to make mistakes in the understanding of the terms of reflux symptoms. Among GERD patients, all the terms of reflux symptoms could be understood accurately. Compared with that of non-GI physicians, GI physician had a higher accuracy in the understanding of the term regurgitation (*P* < 0.05). Pictograms denoting reflux symptoms could be understood accurately in all four groups. A sample from the general population showed that the recognition of the pictogram was more accurate than the recognition of the terms.

**Conclusions:**

Pictograms could help ordinary people who do not have medical education to understand reflux symptoms more accurately in China. Compared with abstract terms, pictograms could be useful for epidemiological studies and diagnosis of GERD in the community.

## 1. Introduction

Gastroesophageal reflux disease (GERD) is defined as the presence of symptoms related to acid reflux into the esophagus and includes heartburn, regurgitation, and esophageal mucosal damage caused by the abnormal reflux of gastric contents into the esophagus [1]. GERD is one of the most common disorders of the gastrointestinal tract [2]. The prevalence of GERD varies from 2.5% to 33.1% depending upon the region of the world [3]. Although the prevalence of GERD in Asia (2.5–7.8%) is still much lower than that in Western countries (8.8–27.8%) [3], its prevalence has steadily increased in the recent years [4–6]. Various risk factors have been shown to be associated with high prevalence of GERD including body weight, genetic factors, pregnancy nutrition, alcohol consumption, sleeping position, and increased fat in diet [1, 7–9]. Nevertheless, because most people in Asia do not speak English and because the original diagnosis systems of GERD are in English, translation may pose a barrier for the right understanding of GERD symptoms in Asia. Therefore, this could be another reason for the low prevalence in Asia, that is, low recognition because of misunderstanding of GERD symptoms by people and community physicians. Based on the impacts of GERD on the quality of life [10, 11], timely accurate diagnosis and adequate treatments should improve the quality of life of patients with GERD and reduce medical expenses from GERD for patients in non-English-speaking countries. In addition, in China, outpatient clinics are overburdened [12, 13], highlighting the need for fast recognition of the symptoms of GERD.

The diagnosis of GERD is based on the presence of symptoms of gastroesophageal reflux [1, 8, 9]. Gastroesophageal reflux symptom score systems (including reflux disease questionnaire (RDQ), GERD impact scale (GIS), and GERD questionnaire (GERDQ)) play key roles in the diagnosis and evaluation of GERD [14, 15]. The description of the reflux symptoms are the mainstay of the entire diagnosis systems mentioned above, but the terms describing these symptoms were expressed originally in English. Because of language and cultural differences, different ethnic groups may have different perceptions and expression of their symptoms. In non-English-speaking countries, patients have to hear, see, and discriminate their specific situation using terms denoting reflux symptoms translated from English. For ordinary people, this may be confusing without help.

It is well known that compared with abstract terms, pictograms are intuitive and are more easily interpreted and perceived, but whether or not pictograms could help the accurate recognition of reflux symptoms in non-English-speaking countries is still unknown. Therefore, the aim of the present study was to compare the difference of recognition of classic reflux symptoms between pictograms and terms in China.

## 2. Materials and Methods

### 2.1. Study Design and Subjects

This was a prospective study that was carried out from September 2012 to December 2014 at the Tianjin Medical University General Hospital. It consisted two separate protocols. In the first protocol, 4 groups were included: group 1 (physicians working at the Gastroenterology Clinic), group 2 (physicians at the other departments (nephrology, neurology, cardiology, endocrinology, and rheumatology) recruited using posters), group 3 (healthy people without medical education recruited from Tianjin University using posters), and group 4 (GERD patients identified from the endoscopy database of the Gastroenterology Clinic of the Tianjin Medical University General Hospital. For groups 1–3, exclusion criterion was (1) education level lower than middle school, (2) any complaint of symptoms of GERD, (3) history of any digestive disease, (4) inability to understand Chinese, or (5) cognitive impairments. For group 4, exclusion criterion was (1) without erosion of the esophageal mucosa, (2) education level lower than middle school, (3) inability to understand Chinese, or (4) cognitive impairments.

The second protocol of the study was to test the recognition of the pictograms in a sample from the general population. A group of healthy people (*n* = 100) was recruited from the Tianjin Medical University General Hospital Physical Examination Center by poster advertisement. The inclusion criteria were (1) 18–65 years of age, (2) education above primary school, and (3) no history of digestive system diseases. The exclusion criterion was (1) refused the study, (2) abnormal vision, or (3) nervous system disease that affects the ability to understand.

The present study was approved by the Institutional Review Board and Ethical Committee of the Tianjin Medical University General Hospital. All subjects provided a written informed consent before enrolment.

### 2.2. Pictograms

**Figure 1:**
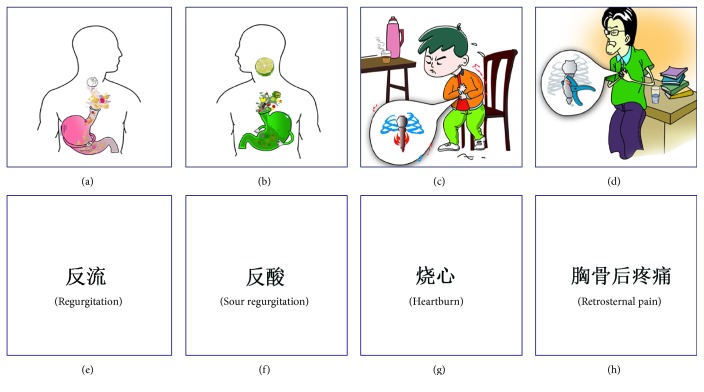
Pictograms denoting the classic gastroesophageal reflux symptoms. Pictograms denoting reflux symptoms were created by the joint efforts of an artist and a gastroenterologist specialized in GERD. (a) Pictogram denoting regurgitation, the feeling of the flowing back of stomach contents. (b) Pictogram denoting sour regurgitation, the feeling of acidic stomach content flowing into the pharynx and mouth. (c) Pictogram denoting heartburn, the feeling of burn behind the breastbone. (d) Pictogram denoting retrosternal pain, the feeling of pain behind the breastbone and above the xiphoid. (e)–(h) Cards with the terms of the GERD symptoms.

Pictograms denoting classic reflux symptoms (sour regurgitation, heartburn, retrosternal pain, and regurgitation) were created by the joint efforts of an artist and a gastroenterologist specialized in GERD (Figure 1). Eight cards printed with terms of reflux symptoms or pictograms denoting these symptoms were prepared. The shape, color, and size of the cards were all the same. There was only one term or one pictogram on each card. All the cards were placed in a black-colored box.

During the study, the subject was asked to go in a quiet room and to sit down before three gastroenterologists specialized in GERD. After the subject was made comfortable, he or she was informed that the following test was about symptoms of digestive system disease and asked to take a card from the box. Then, the subject was asked to have a look at the card and to tell the meaning of the word or pictogram on the card within 30 s and then to put the card in a basket beside the box. The procedure was repeated until the last card was taken from the box. After the test was finished, the cards were shuffled and put back in the box. Only one subject was allowed to have the test at one time. The time to provide an answer was noted. The attempt was considered failed after 30 s.

The definition provided by the subject was judged by the three gastroenterologists, who were blinded to grouping. If there was a disagreement, the final decision was made upon mutual agreement through discussion. Only the accurate definition of the meaning of the reflux symptom was regarded as right. The accurate meaning of reflux symptoms were (1) heartburn was denoted as having a burn feeling behind the breastbone, (2) regurgitation was denoted as the feeling of fluids flowing back from the stomach into the esophagus, (3) sour regurgitation was denoted as the feeling of acidic stomach content flowing into the pharynx and mouth, and (4) retrosternal pain as the feeling of pain behind the breastbone and above the xiphoid.

At the end of the test, all the subjects were also asked to evaluate the use of pictogram on the understanding of reflux symptoms as great help, help, and no help.

The group of 100 individuals from the general population was randomized to the terms and pictograms groups. The individuals in the terms group had to associate the pictograms to the terms, while the individuals in the pictograms group had to associate the terms to the pictograms. The time of association was evaluated.

### 2.3. Statistical Analysis

Normally distributed variables were presented as mean ± standard deviation and analyzed using ANOVA with the Tukey's post hoc test. Nonnormally distributed variables were presented as median (range) and analyzed using the Kruskal-Wallis test with the Mann–Whitney post hoc test. Categorical data were presented as frequencies and analyzed using the chi-square test or Fisher's exact test, as appropriate. In the group of 100 individuals from the general population, accuracy was tested using the chi-square test. All analyses were carried out using SPSS 16.0 (IBM, Armonk, NY, USA). Two-sided *P* values <0.05 were considered statistically significant.

## 3. Results

### 3.1. Characteristics of the Subjects

In the first protocol, this study included physicians working at the Gastroenterology Clinic (*n* = 28), physicians working at the other departments (*n* = 30), and healthy people without medical education (*n* = 34). Forty-five GERD patients (course of disease of 7.1 (range: 2, 22) months) were also included. There was no significant difference in age, gender, and educational level among the groups (all *P* > 0.05) (Table 1).

### 3.2. Recognition of Symptom Terms

**Figure 2:**
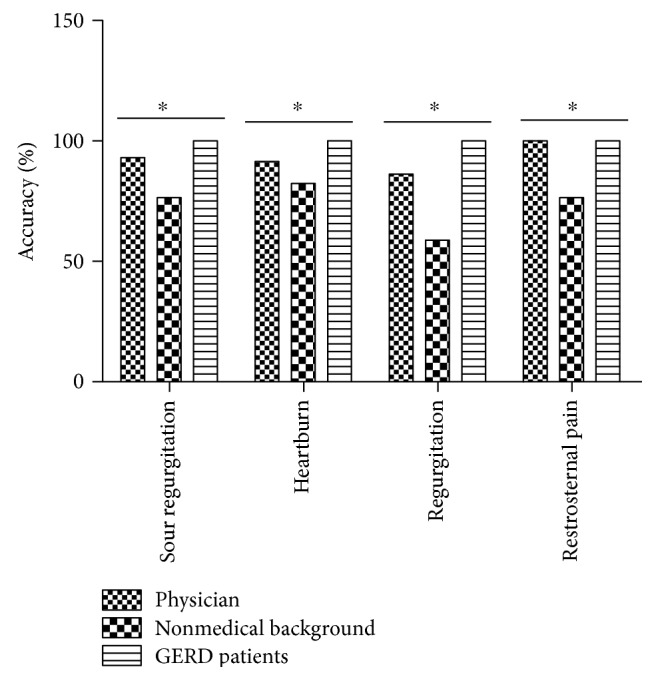
Difference in the accuracy of the understanding the terms of reflux symptoms among the groups. ^∗^*P* < 0.05 among the groups, chi-square test.

Compared with the physicians, healthy people without medical education tended to make mistakes in the understanding of the terms of reflux symptoms. Among them, 58.8% (20/34) had difficulty in the understanding of more than two items of the reflux symptom terms. The accuracy of understanding the term of sour regurgitation was 93.1% (54/58), 76.5% (26/34), and 100% (45/45) for physicians, subjects without medical background, and GERD patients, respectively (chi-square test, *P* = 0.001). For heartburn, the proportions were 91.4% (53/58), 82.4% (28/34), and 100% (45/45), respectively (chi-square test, *P* = 0.017). For regurgitation, the proportions were 86.2% (50/58), 58.8% (20/34), and 100% (45/45), respectively (chi-square test, *P* < 0.0001). And for retrosternal pain, the proportions were 100% (58/58), 76.5% (26/34), and 100% (45/45), respectively (chi-square test, *P* < 0.0001) (Figure 2).

**Figure 3:**
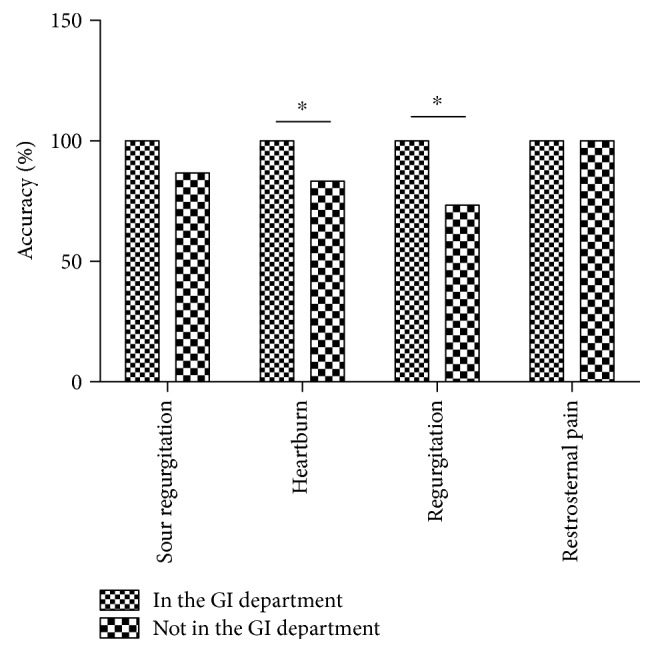
Accuracy of understanding classic reflux symptoms in physicians. ^∗^*P* < 0.05 compared with that in the GI department, Fisher's exact test.

Compared with non-GI physicians, GI physician had a higher accuracy in the understanding of the terms regurgitation (100% (28/28) versus 73.3% (22/30), Fisher's exact test, *P* < 0.0001) and heartburn (100% (28/28) versus 83.3% (25/30), Fisher's exact test, *P* = 0.03). There was no difference on the recognition of the terms sour regurgitation (100% (28/28) versus 86.7% (26/30), Fisher's exact test, *P* = 0.06) and retrosternal pain (100% (30/30) versus 100% (28/28), Fisher's exact test, *P* = 1.00) (Figure 3).

### 3.3. Recognition of Symptom Pictograms

**Figure 4:**
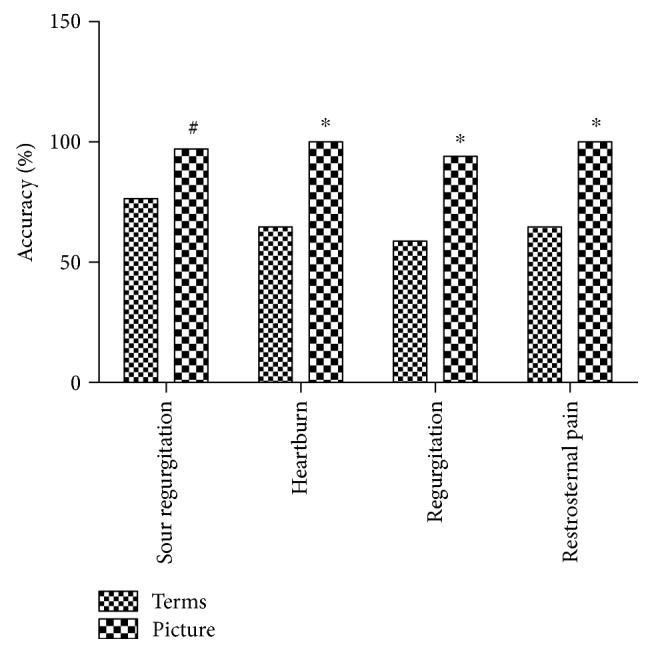
Influence of pictograms on understanding classic reflux symptoms in people without medical background in community. Fisher's exact test: ^∗^*P* < 0.05, compared with the terms; ^#^*P* < 0.01 compared with the terms.

Pictograms denoting reflux symptoms could be understood accurately in all 4 groups. In healthy people without medical education, the accuracy of understanding terms or pictograms denoting sour regurgitation was 76.5% (26/34) and 97.1% (33/34) (Fisher's exact test, *P* = 0.003), 64.7% (22/34) and 100% (34/34) for heartburn (Fisher's exact test, *P* < 0.001), 58.8% (20/34) and 94.1% (32/34) for regurgitation (Fisher's exact test, *P* = 0.002), and 64.7% (22/34) and 100% (34/34) for retrosternal pain (Fisher's exact test, *P* = 0.004) (Figure 4).

**Figure 5:**
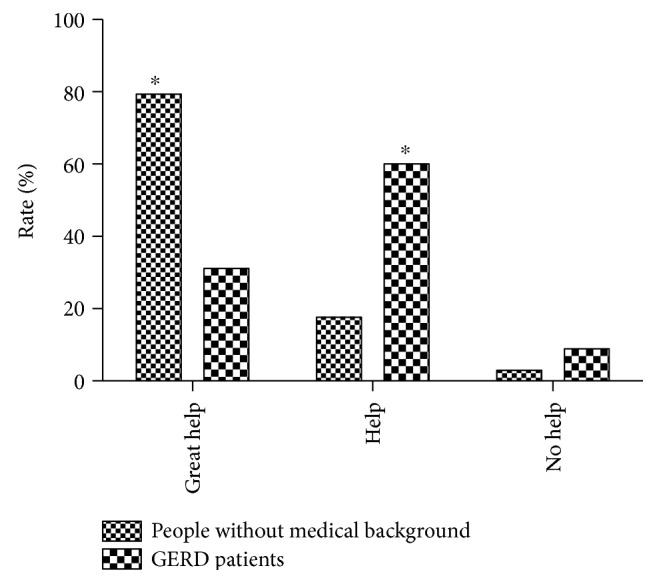
Evaluation of pictograms on the understanding of classic reflux symptoms. ^∗^*P* < 0.05 compared with the GERD patients, Fisher's exact test.

Compared with GERD patients, more people without medical education regarded the pictograms as being of great help (79.4% (27/34) versus 31.1% (14/45), Fisher's exact test, *P* < 0.001). More GERD patients regarded pictograms as being of help (60.0% (27/45) versus 17.7% (6/34), Fisher's exact test, *P* < 0.001). Only few people in both groups regarded pictograms as being of no help (2.9% (1/34) and 8.9% (4/45)) (Figure 5). For GI physicians, pictograms were thought to be of great help (21.43%, 6/28), of help (57.14%, 16/28), and not helpful (21.43%, 6/28) compared with 26.67% (8/30), 40.00% (12/30), and 33.33% (10/30), respectively, by non-GI physicians (Fisher's exact test, all *P* > 0.05).

### 3.4. Validation Using the Sample from the General Population

There was no difference in age (42.8 ± 11.6 versus 43.6 ± 11.2, *P* = 0.72), gender (males, 58% versus 52%, *P* = 0.55), and level of education (primary school, middle school, college: 28%, 52%, 20% versus 26%, 48%, 26%, *P* = 0.78) between the terms group and the pictogram group.

Recognition of each symptom was better in the pictogram group compared with the term group (all *P* < 0.05). The time was also shorter in the pictogram group compared with the term group (all *P* < 0.0001) (Table 2).

## 4. Discussion

GERD is a widespread disease commonly seen in clinics in Western countries [16]. The prevalence of GERD is increasing in developing countries and poses a heavy burden on the medical system [4–6]. Several strategies have been developed to diagnose GERD without the need of invasive methods such as endoscopy, pH meter, and impendence monitoring. Questionnaires are one of these tools and allow an objective assessment of symptoms [14, 15]. These questionnaires usually include a series of questions to assess severity, frequency, related phenomena, and sometimes quality of life. Advantages of the questionnaires include that they can be self-administered, can be used as screening tools, are inexpensive, and can be applied to any subject suffering from GERD. The mainstay of these questionnaires is various reflux symptoms, especially classic reflux symptoms such as sour regurgitation, heartburn, regurgitation, and retrosternal pain [17].

To effectively use these questionnaires, easy, quick, and accurate understanding of reflux symptoms by patients in a clinic or subjects in epidemiological studies is required. Nevertheless, a main obstacle to the use of these questionnaires in China is that the original versions of these questionnaires are in English. To be used in non-English-speaking countries, these questionnaires can be translated into various languages [18, 19]. These translated versions can be certainly understood accurately by well-educated specialists in these countries, but difficulties may arise for patients and people of the general population. A big issue is the misunderstanding of some abstract words, especially medical terms. This poses an obstacle for the effective use of questionnaires for GERD in non-English-speaking countries [20]. For instance, in the use of Chinese GERDQ, since there is no direct translation of the word “heartburn” in the Chinese language, a burning pain or discomfort behind the breastbone is used as the definition of heartburn. Despite this explanation, heartburn is only present in 50% of Chinese patients with GERD, which is considerably lower than in the Western series [18, 21]. A study on the frequency of symptoms and complications of GERD in different ethnic groups also found that the term “heartburn” was understood by only 35%, 54%, and 13% of Whites, Blacks, and East Asians, respectively [22]. This suggests that the abstract term of reflux symptom is liable to be misinterpreted. Different from a previous report in China [18], the term heartburn was understood accurately (82.4%) in the present study, which could be due to the use of pictograms and public education on GERD in China.

The process of comprehension of abstract words is sophisticated [23]. Compared with abstract words, pictograms such as traffic signs can make it easier for ordinary people to interpret and perceive information. In fact, Chinese characters belong to ideographs which have the feature-like graphic and are based on pictogram. Nevertheless, it is still hard to tell the meaning of a Chinese word intuitively. It was speculated that using pictograms, the abstract meaning of reflux symptoms expressed in words could be transformed to information which could be easily understood by ordinary people. In the present study, pictograms denoting reflux symptoms could be understood accurately both by physicians and by people without medical background. In addition, as expected, pictograms could bring a higher accuracy of understanding reflux symptoms compared with abstract terms in healthy people without medical education. The rates were also high for GERD patients. This accuracy was higher than in a previous study in which only 49.7% (92/185) of GERD patients regarded the reflux symptoms as easily comprehensible [18]. A possible reason for this discrepancy was that almost all GERD patients in the present study had been diagnosed as GERD by endoscopy and explained the symptoms of gastroesophageal reflux by a gastroenterologist somewhere during their management.

Despite the need to create a real questionnaire using these pictograms and to validate this questionnaire, the present study suggests that using pictograms could be useful for the easy, rapid, and inexpensive diagnosis of symptoms of GERD. Since this questionnaire would be filled by the patients without the help of the physician, it could help diminish the pressure on the healthcare system, which is a major problem in China [24, 25].

Of course, the present study is not without limitations. First, the sample size was small and larger cohorts are necessary to validate these results. Secondly, patients with nonerosive reflux disease should also be included. Finally, because of the nature of the groups and the testing process, strict randomization was not possible.

In conclusion, pictograms could help ordinary people who do not have medical education to understand reflux symptoms more accurately in China. Compared with abstract terms, pictograms could be useful for epidemiological studies and diagnosis of GERD in the community.

## Figures and Tables

**Table 1:** Characteristics of the subjects.

	GI physicians	Non-GI physicians	Healthy people	GERD patients	*P*
*n*	28	30	34	45	—
Age (years)	30.5 ± 3.2	30.0 ± 3.4	29.8 ± 4.0	29.1 ± 3.6	0.437
Gender (M/F)	10/18	14/16	18/16	24/21	0.468
Education (college/middle school)	28/0	30/0	32/2	41/4	0.173

**Table 2:** Comparison of accuracy and timing between the two groups of the general population.

	Picture group	Term group	*P*
*Accuracy (%)*			
Sour regurgitation	96.0% (48/50)	74.0% (37/50)	0.005
Heartburn	94.0% (47/50)	68.0% (34/50)	0.002
Regurgitation	88.0% (44/50)	62.0% (31/50)	0.006
Retrosternal pain	94.0% (47/50)	66.0% (33/50)	0.001
*Time (s)*			
Sour regurgitation	14.8 ± 3.4	24.0 ± 3.5	<0.0001
Heartburn	14.8 ± 2.6	23.8 ± 3.2	<0.0001
Regurgitation	14.5 ± 2.8	24.0 ± 3.2	<0.0001
Retrosternal pain	14.2 ± 2.4	24.3 ± 3.3	<0.0001
